# Neovaginal Human Papilloma Virus–Related Squamous Cell Carcinoma in a Transgender Woman

**DOI:** 10.1001/jamanetworkopen.2024.2537

**Published:** 2024-03-15

**Authors:** Susan M. Lang, Ravali A. Reddy, Malte Renz

**Affiliations:** 1 Gynecologic Oncology Division, Stanford University, Palo Alto, California

## Abstract

This case series discusses a human papilloma virus (HPV)–related neovaginal squamous cell carcinoma in a transgender woman and the need for formal gynecologic screening recommendations.

## Introduction

Neovaginal squamous cell carcinomas (SCCs) can occur in transgender women following gender-affirming surgery (GAS). Although rare, with only 5 SCC cases reported,^1,2,3,4^ the number of neovaginal SCCs may increase with improved access to GAS. The number of GASs tripled between 2016 and 2020.^5^ Creating a neovagina involves inversion of penile-scrotal skin. This unscreened reproductive tissue with high human papilloma virus (HPV) prevalence may be at increased risk of developing SCC once heterotopic and inverted,^1^ but formal recommendations regarding surgical follow-up examinations, screening, and treatment are missing.

## Methods

In this case series, a transgender woman in her 60s was referred to gynecologic oncology for a newly diagnosed HPV-related neovaginal SCC. Her GAS, performed in 2014, included penectomy, bilateral orchiectomy, and neovaginal creation by penile-scrotal skin inversion. The Stanford University institutional review board waived review of this study as exempt. Written informed consent was obtained from the patient. This review followed the Appropriate Use and Reporting of Uncontrolled Case Series in the Medical Literature guideline.

## Results

In 2022, the patient presented with urinary incontinence and vulvovaginal discomfort for 3 months. Biopsies of verrucous tissue obliterating the vagina were positive for invasive SCC and HPV by in-situ hybridization. Magnetic resonance imaging of the pelvis showed a 2.4 × 2.1 × 6.0–cm neovaginal mass invading the prostate and puborectalis muscles. Positron emission tomography/computed tomography (PET/CT) showed intense fluorodeoxyglucose (FDG) uptake along the entire circumference and length of the neovagina and in inguinal and obturator lymph nodes bilaterally (Figure). Since this cancer developed in invaginated tissue, the tumor board consensus was for primary chemoradiation with external beam radiation and concurrent weekly cisplatin followed by 4 fractions of high-dose rate brachytherapy. Posttreatment PET/CT 3 months later showed a decrease in size (1.6 × 1.2 × 1.4 cm) and FDG avidity consistent with partial treatment response.

**Figure.**
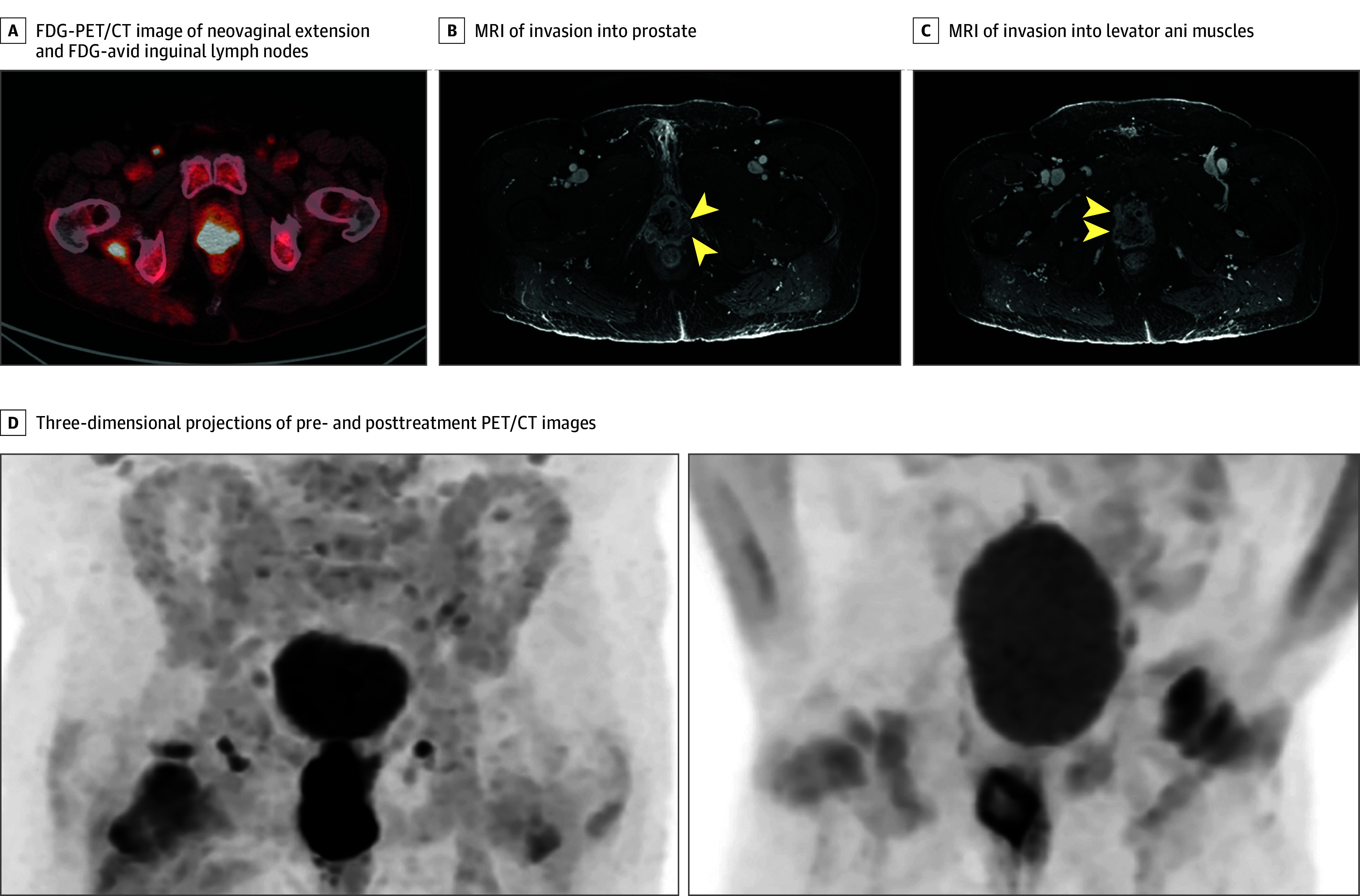
Imaging of Human Papilloma Virus–Related Neovaginal Squamous Cell Carcinoma Extent Arrowheads in panels B and C indicate invasion into the prostate and levator ani bilaterally. FDG-PET/CT indicates fluorodeoxyglucose–positron emission tomography/computed tomography; MRI, magnetic resonance imaging.

## Discussion

Given improved access and increased GAS numbers, this case of locally advanced neovaginal SCC highlights the absence of formal recommendations regarding surgical follow-up, screening for dysplasia and HPV, and treatment of neovaginal SCC. Although the American Society of Colposcopy and Cervical Pathology advises transgender men who retain their cervix should follow the same guidelines as cisgender women, there are no recommendations for transgender women with neovaginas.

The absence of recommendations for transgender women may be due to the lack of data and research, which also limits this case report. Development of centralized cancer registries can help collate clinicopathologic information and HPV, dysplasia, and SCC incidence and outcomes.

Regular follow-up pelvic and speculum examinations can be performed in transgender care centers to assess neovaginal patency and potential mucosal pathology. Such a structured framework may help detect dysplasia early.

Vaginal cancers comprise 2% of all gynecologic malignant neoplasms, with an HPV prevalence of 75% vs 99.8% in cervical cancers. However, HPV prevalence is high (24%-74%) and not routinely screened for in persons assigned male at birth. Reported prevalence of high-risk HPV in sexually active transgender women is 8% to 20% and of HPV-related dysplasia, 10%.^6^ Whether cytologic screening results from the neovagina are comparable with those from native cervices is unclear; HPV testing might be more sensitive and cost-effective. Formalized recommendations could include routine preoperative screening of the penile-scrotal skin used for the neovagina to help risk-stratify postoperative examinations.

Treatment of neovaginal SCC may follow cervicovaginal cancer treatment since the HPV-related SCC arises in invaginated tissue and is radiation sensitive. Short- and long-term outcomes of pelvic radiation include inflammation and scarring of neovaginal tissue. The fact that many transgender women retain the prostate does not preclude them from gynecologic oncology care.

## Conclusions

This case series indicates a need for improved data collection through centralized cancer registries. Guideline recommendations for transgender women may help promote physical health and broaden health equity with the inclusion of a gender-diverse population.

## Notes

Supplement.
Data Sharing Statement
