# Supplementary material for: Neovaginal Human Papilloma Virus–Related Squamous Cell Carcinoma in a Transgender Woman
Source: JAMA Netw Open. 2024 Mar 15;7(3):e242537. doi: 10.1001/jamanetworkopen.2024.2537 (PMC10943404; doi:10.1001/jamanetworkopen.2024.2537)
Supplement: Supplement. — Data Sharing Statement [file jamanetwopen-e242537-s001.pdf]

Data Sharing Statement

Lang. Neovaginal Human Papilloma Virus–Related Squamous Cell Carcinoma in a Transgender Woman. *JAMA Netw Open*. Published March 15, 2024. doi:10.1001/jamanetworkopen.2024.2537

Data
Data available: No

Additional Information
Explanation for why data not available: De-identified data can be made available upon request
